# Discovery of the molecular mechanisms of the novel chalcone-based *Magnaporthe oryzae* inhibitor C1 using transcriptomic profiling and co-expression network analysis

**DOI:** 10.1186/s40064-016-3385-9

**Published:** 2016-10-22

**Authors:** Hui Chen, Xiaoyun Wang, Hong Jin, Rui Liu, Taiping Hou

**Affiliations:** 1 Key Laboratory of Bio-Resource and Eco-environment of Ministry of Education, College of Life Sciences, Sichuan University, Chengdu, 610064 China; 2 State Key Laboratory of Oral Disease, West China School of Stomatology, Sichuan University, Chengdu, 610041 China

**Keywords:** *Magnaporthe oryzae*, Transcriptome, Gene co-expression network, Molecular docking

## Abstract

**Background:**

In our previous studies, we discovered a series of chalcone-based phytopathogenic fungus inhibitors. However, knowledge of their effects, detailed targets and molecular mechanisms in *Magnaporthe oryzae* (*M. oryzae*) remained limited.

**Methods:**

To explore the expression and function of differentially expressed genes in *M. oryzae* after treatment with compound C1, we analyzed the expression profile of mRNAs using a microarray analysis and GO, KEGG and WGCNA analysis, followed by qRT-PCR and Western blots to validate our findings.

**Results:**

A total of 1013 up-regulated and 995 down-regulated mRNAs were differentially expressed after *M. oryzae* was treated with C1 compared to those of the control samples. Among these, cytochrome P450, glycylpeptide N-myristoyltransferase (NMT) and peroxisomal membrane protein 4 were identified as the most significant DEGs and were validated by experiments.

**Conclusion:**

In conclusion, our study suggests that the combination of transcriptomic microarray, bioinformatics analysis and weighted gene co-expression networks can be used to predict potential therapeutic targets and to map the pathways regulated by small molecular natural product-like drugs.

**Electronic supplementary material:**

The online version of this article (doi:10.1186/s40064-016-3385-9) contains supplementary material, which is available to authorized users.

## Background

Fungal infections are one of the most important phytopathogens that affects agricultural output (Lu et al. 2014; Moghaddam et al. 2015; Moreira et al. 2015). Some fungicides have been used to control these diseases and protect livestock, but their use has led to toxic chemical accumulation in the environment, causing servere environmental and public health problems (Kim et al. 2014; Wang et al. 2015; Wu et al. 2014; Lopez et al. 2006, 2011; Svetaz et al. 2007). Of the phytopathogenic fungi, *Magnaporthe oryzae* is one of the most common rice blast pathogens; *M. oryzae* is resilient to environmental stresses such as changes in nutrients, illumination and temperature (Duan et al. 2014; Hao et al. 2012a, b). *M. oryzae* has gradually become resistant to existing fungicides; consequently, it is essential to discover novel, environmentally friendly compounds with high antifungal activity and clear molecular mechanisms of action (Xu et al. 2015; Dong et al. 2015; Wang et al. 2014; Chen et al. 2013).

**Fig. 1:**
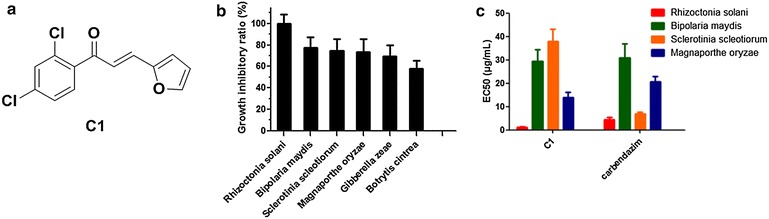
Chemical structure and inhibitory effects on the phytopathogens of chalcone-based compound C1: **a** the chemical structure of **C1**; **b** the growth inhibitory ratio of C1 on a panel of phytopathogens at 40 μg/mL; **c** the EC_50_ values of **C1** on several phytopathogens

**Scheme 1:**
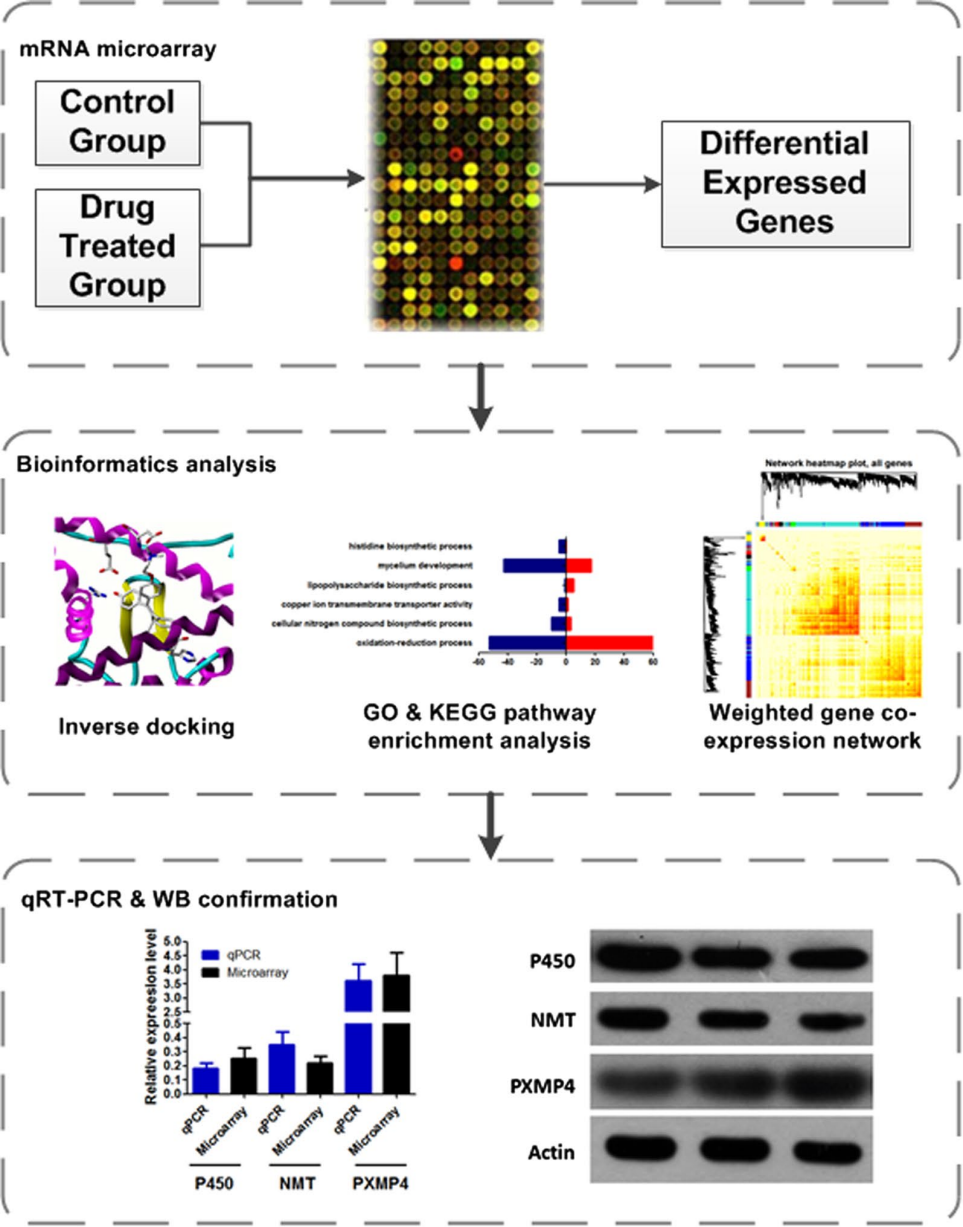
Flowchart of the methods. The scheme includes three steps. Firstly, differentially expressed mRNAs in drug-treated *M. oryzae* compared with the control group were identified via microarray analysis. Then, bioinformatics analysis, including GO and KEGG pathway enrichment, inverse docking and WGCNA network, were performed for speculating the correlation between potential targets and DEGs. Subsequently, we utilized qRT-PCR and Western blot to validate the microarray results

Past research has detailed the antifungal activities, the structure–activity relationships (SARs) and the inhibitory capacity of the fungal cell wall synthesis pathway for a series of chalcone derivatives (Lopez et al. 2001, 2006, 2011; Svetaz et al. 2004, 2007; Boeck et al. 2005). In our previous study of the anti-phytopathogenic fungus capacities of chalcone derivatives, we screened a large number of compounds and provided information on the SARs of these compounds (Ren et al. 2015; Zhang et al. 2011; Teng et al. 2010; Yu et al. 2009; Liu et al. 2009; Jin et al. 2009). Of these chalcone derivatives, 1-(2′,4′-dichlorophenyl)-3-(2-furyl)-2-propen-1- one (compound C1, Fig. 1a) is one of the most potent compounds with a broad antifungal spectrum of phytopathogens. Zacchino et al. reported C1 could inhibit β(1,3)-glucan synthase and chitin synthase in *Saccharomyces cerevisiae* (Svetaz et al. 2004; Lopez et al. 2001); however, its antifungal capacity toward *M. oryzae*, as well as its molecular mechanisms, remain unknown so far. In the current study, we integrate results from a transcriptomic microarray, a bioinformatics analysis, a gene co-expression network analysis and experimental validations to profile the potential targets and related pathways of chalcone-based C1 treatment in *M. oryzae* (Scheme 1). Our results revealed that the mRNA expression profile was dramatically different between C1-treated and control groups. Cytochrome p450 and N-myristoyltransferase (NMT) might bind to C1 and be downregulated following C1 treatment. Next, we performed a weighted gene co-expression network analysis of DEGs and found that cytochrome p450, NMT and PXMP4 were involved with the hub genes, indicating that the mechanism of compound C1 might involve multiple targets and multiple pathways. Finally, we performed qRT-PCR and western blot experiments to verify the results of the microarray and bioinformatics analysis.

## Methods

### In vitro antifungal assay

The antifungal activity of compound C1 against *B. maydis, S. scleotiorum, M. orzae, G. zeae and B. cintrea* was investigated by measuring the inhibitory effects of radial growth in a petri dish with agar medium. Briefly, compound C1 was dissolved in DMSO, diluted with a 1 % Tween solution to the appropriate concentration and added to a glucose/potato/water agar medium to reach the final concentration. The medium was then poured into 9.0 cm diameter sterile Petri dishes, and a mycelial disk (0.5-cm diameter) cut from the growing culture was placed in the center of the agar plate. The inhibitory effects of C1 on radial growth was measured after several days. Inhibitory growth ratios were calculated as the percentage of the inhibition of radial growth relative to the control group.

### RNA isolation and labeling

Total mRNA was extracted from *M. orzae* samples using TRIzol, according to the manufacturer’s instructions. A NanoDrop ND-2000 was used to assess the concentration and quality of RNA before the extracted RNA was denatured for agarose gel electrophoresis. The purified RNA was amplified and transcribed into fluorescent cRNA after the removal of rRNA according to Agilent’s Quick Amp Labeling protocol.

### Microarray analysis

Microarray analysis was performed by Boao Bio-tech, Beijing, China. Briefly, the labeled cRNAs were hybridized onto the 4 × 44 K Agilent Microarray (Arraystar, Rockville, MD) at 65 °C for 17 h. An Agilent Microarray Scanner G2565BA was used to collect the hybridization images. The transcriptomic data extraction and analysis were performed using the Agilent Feature Extraction package and GeneSpring GX v11.5.1 software, respectively.

### Inverse docking

The compound C1 was submitted to various web-based inverse docking servers: TarFisDock (Gao et al. 2008; Li et al. 2006), DRAR-CPI (Luo et al. 2011), and PharmMapper (Wang et al. 2016; Liu et al. 2010). These web servers selected the known target proteins within their collections to profile the scaffolds that had potential binding affinity.

The Target Fishing Dock (TarFisDock) identified the potential target proteins of submitted molecules in the Potential Drug Target Database using the DOCK 4.0 molecular docking program (Kang et al. 2004; Ewing et al. 2001); only the top 2 % of potential targets were considered for further study. The PharmMapper server is a structure-based pharmacophore approach that accelerates the screening of putative binding targets for small molecular drugs (Wang et al. 2016). The DRAR-CPI server predicts adverse drug reactions and therapeutic indications for small molecular drugs based on the interaction profile of molecules towards their targets (Iyer et al. 2015; Chen 2014).

### GO & KEGG enrichment analysis

Gene Ontology (GO) analysis is an annotation set including gene descriptions and gene product attributes for many organisms (http://www.geneontology.org) (Chicco and Masseroli 2016; Anney et al. 2011; Harris et al. 2004; Ashburner et al. 2000). Gene ontology has three components: cellular components, biological processes and molecular functions. The overlaps between the lists of DEGs were detected by Fisher’s exact test. P-value denotes the significance of a GO term enrichment in DEGs clusters and/or pathway correlations (P-value < 0.05 was considered significant). In addition, the pathway enrichment was used to map DEGs into KEGG pathways (Ogata et al. 1999; Ogata et al. 1998).

### Weighted gene co-expression network analysis (WGCNA)

WGCNA is a statistical tool to cluster genes that have a similar expression pattern across a group of samples (Malki et al. 2013; DiLeo et al. 2011; Langfelder and Horvath 2008). The input data for the WGCNA were the normalized gene expression values for each sample. First, all available samples from each groups were collected to identify modules that had different expression patterns. Next, a soft threshold was assigned to create networks with a scale free topology, using the method developed by Horvath et al. After the networks were built, many gene modules with similar expression patterns were created, and the eigengenes of these modules were calculated. Finally, correlations between these eigengenes and the factor of interest were calculated.

### Quantitative real-time PCR (qRT-PCR)

Total mRNA was extracted from *M. orzae* using TRIzol (Invitrogen), according to the manufacturer’s protocols. mRNAs were then converted into cDNA using a Fermentas RT kit. qRT-PCR was performed in a total reaction volume of 25 μL (including 12.5 μL of SYBR Premix Ex Taq (2×), 2 μL of cDNA, 1 μL of forward primer (10 μM), 1 μL of reverse primer (10 μM), 0.5 μL of ROX Reference Dye II (50×), and 8 μL of double-distilled water). The amplification conditions were as follows: 10 min at 95 °C to initiate denaturation; 40 cycles of 5 s at 95 °C, 30 s at 63 °C, and 30 s at 72 °C; and a final extension for 5 min at 72 °C. The amplification efficiency was evaluated using standard curve fitting. All samples were normalized to actin, and the experiment was performed with three duplicates.

### Western blot analysis

Briefly, the total protein of C1-treated *M. orzae* was extracted with RIPA buffer (SolarBio, Beijing, China), which contained 1 % (v/v) PMSF (SolarBio), 0.3 % (v/v) protease inhibitor (Sigma, St. Louis, MO, USA) and 0.1 % (v/v) phosphorylated proteinase inhibitor (Sigma). Then, the supernatant was collected after centrifugation at 12,000 rpm for 10 min with refrigeration. The concentration of total protein was quantified using a BCA protein assay kit (Pierce, Waltham, MA, USA). The total protein was separated via standard SDS-PAGE gel electrophoresis and then transferred to PVDF membranes. The membranes were further treated with skimmed milk or BSA to block non-specific binding. The primary antibodies were added to PVDF membranes for two hours at room temperature or overnight at 4 °C. Finally, the primary antibodies bound to the membranes were incubated with HRP-conjugated secondary antibodies (Abmart, Shanghai, China). The target proteins were detected using an ECL kit (enhanced chemiluminescence kit, Millipore, Billerica, MA, USA) according to the manufacturer’s instructions.

## Results and discussion

### C1 efficiently inhibited a panel of phytopathogens

The in vitro antifungal results of compound C1 are shown in Fig. 1b, c. The results demonstrate that C1 showed variable degrees of antifungal activity against the tested phytopathogen fungi. The inhibitory ratio at 20 μg/mL clearly demonstrates that C1 exhibited 100 % inhibition against *R. solani* and an inhibition of 60–80 % against *B. maydis*, *S. scleotiorum*, *M. orzae*, *G. zeae* and *B. cintrea*. Moreover, the EC50 index of compound C1 for the tested fungi was in the range of 1.20–37.84 at 20 μg/mL, much lower than that of the positive control compound, carbendazim, on *R. solani* and *M. orzae* (Fig. 1c).

**Fig. 2:**
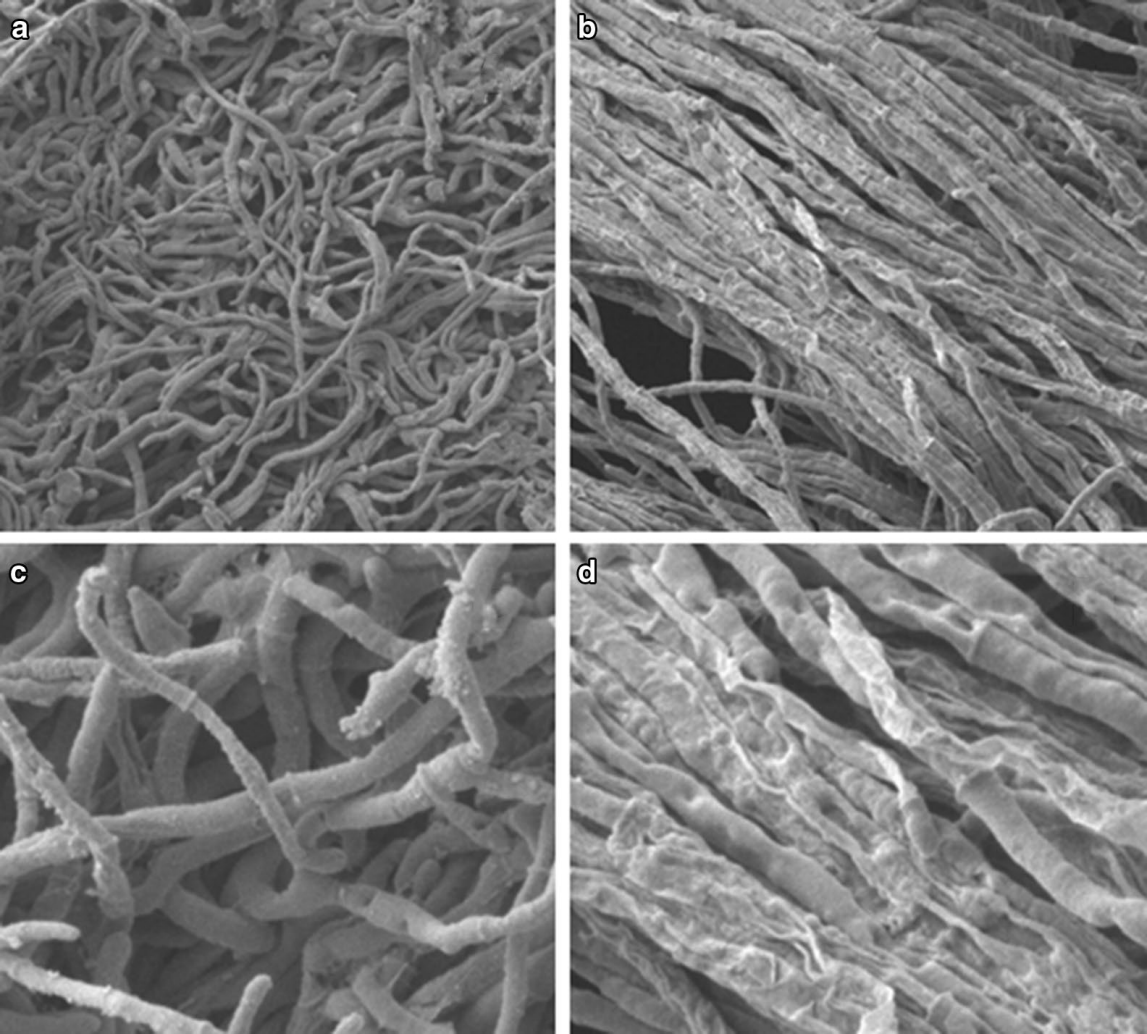
Inhibitory effects of **C1** on *M. oryzae* mycelium observed under SEM: **a** the control group (×1000); **b** the C1 treated group (×1000); **c** the control group (×3000); **d** the C1 treated group (×3000)

In addition to the growth inhibition activity of C1 of *M. oryzae*, the stability of *M. oryzae* mycelium was also affected by treatment with compound C1. As shown in Fig. 2, the scan electronic microscopic (SEM) images show that the mycelium of *M. oryzae* was shrunken and deformed after C1 treatment for 48 h.

### Inverse docking results

**Fig. 3:**
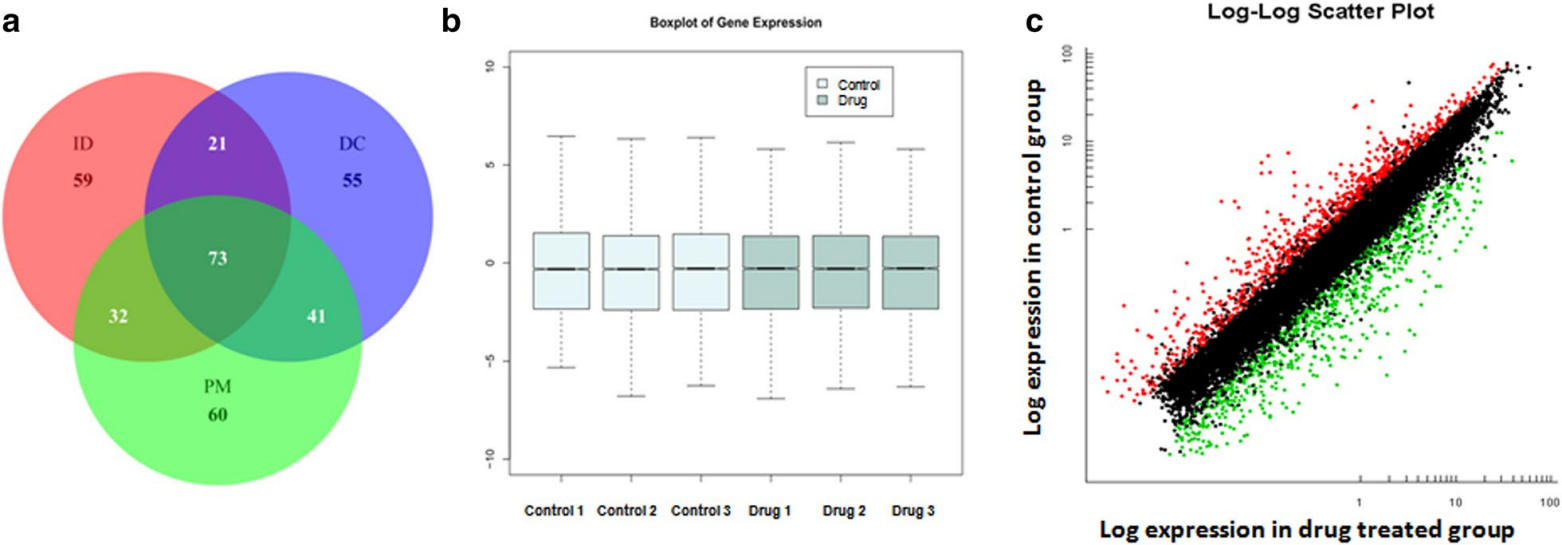
**a** The inverse docked potential targets determined by Tarfisdock (ID), DARA-CPI(DC) and Pharmapper (PM) methods; **b** the *boxplot* of gene expression in different groups; **c** the *scatter plot* of gene expression in different groups

A total of 73 common potential targets for compound C1 were identified using the three web server-based approaches (Fig. 3a). Following assessment for drug-ability using the Potential Drug Target Database (PDTD) (Liu et al. 2010), 185 proteins were predicted to be potential therapeutic targets by Tarfisdock. There were 190 and 206 potential targets predicted by DARA-CPI and PharmMapper, respectively. Among the 73 common targets, four proteins were reported to be involved in fungi’s biological processes. All of the four targets, cytochrome p450, N-myristoyltransferase (NMT), β(1,3)-glucansynthase and chitin synthase were used for further studies.

### Differential expression of genes after C1 treatment determined by transcriptome microarray

We performed a microarray analysis of *M*. *oryzae* after treatment with C1 for 24 h to profile the differentially expressed genes. A total number of 13,448 coding transcripts (mRNA) were detected in C1 treated *M*. *oryzae*; 2008 of which (approximately 9.81 %), were differentially expressed (>2.0-fold, P < 0.05) (Fig. 3b, c; Table 1). Among the 2008 deregulated mRNAs, 1013 mRNAs were upregulated, and 995 mRNAs were downregulated (P < 0.05, Additional files 1, 2). Interestingly, the proportion of upregulated and downregulated mRNAs was approximately 50/50 (Table 1). A scatter plot was used to visualize the mRNAs based on their expression levels (Fig. 3c).

**Table 1:** Numbers of mRNAs differentially expressed after C1 treatment 24 h

Category	Detected genes	Number of DEGs
Total genes	13,448	2008
Up-regulated genes	6013	1013
Down-regulated genes	7435	995

*DEGs* differentially expressed genes

### Bioinformatics analysis

**Fig. 4:**
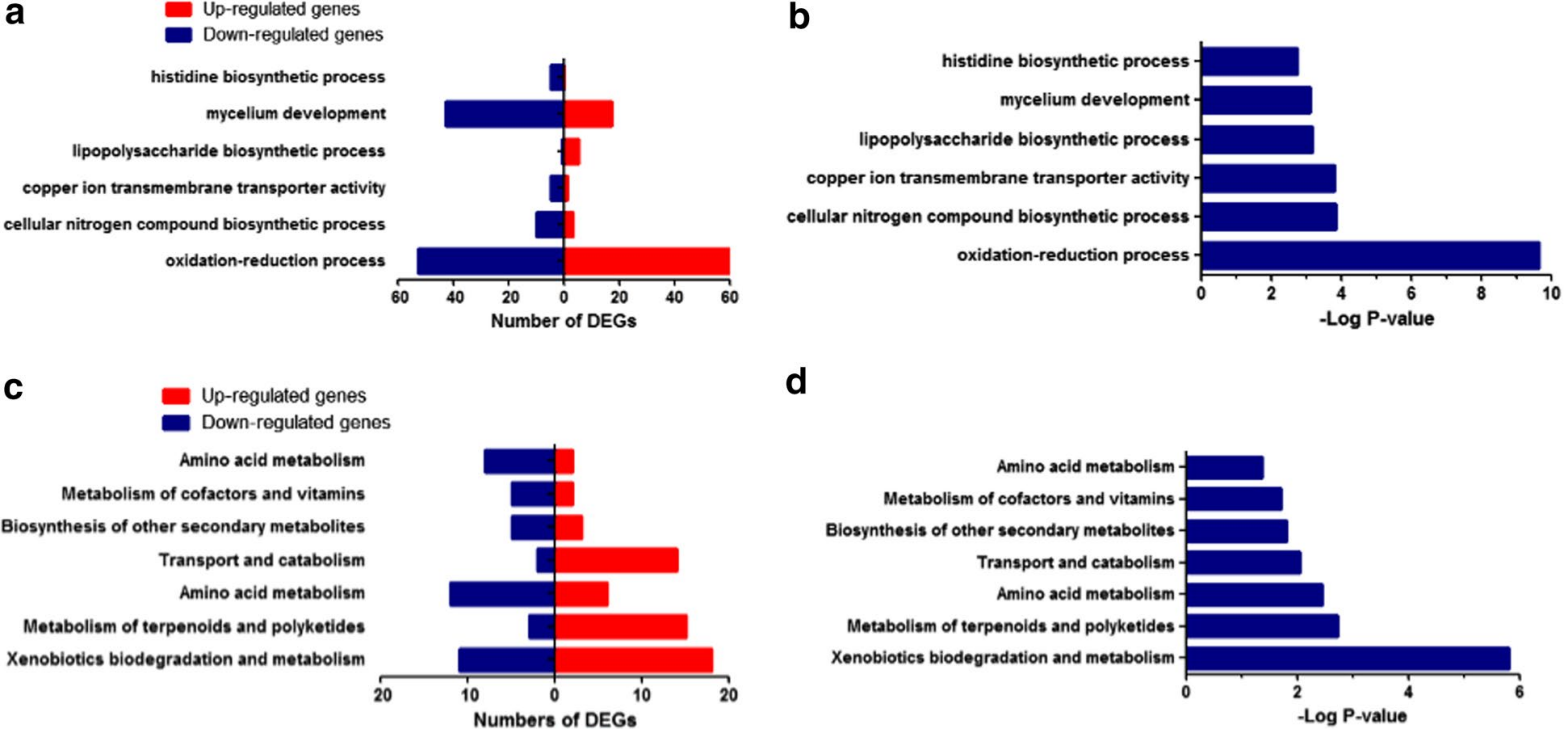
GO enrichment and KEGG pathway analysis of differentially expressed genes in *M. oryzae* according to biological processes. **a** The up-regulated and down-regulated genes in top GO terms enriched among differentially expression genes; **b** the enrichment score (−Log10 P-value) of top GO terms enriched among differentially expression genes; **c** the up-regulated and down-regulated genes in the top enriched pathways among differentially expression genes; **d** the enrichment score of the top enriched pathways among differentially expression genes

It is widely known that the GO enrichment analysis of DEGs (differentially expressed genes) may help provide novel insight into the numerous DEGs with diverse functions. In general, the GO analysis contains three components: biological processes, cellular components and molecular functions. The primarily enriched GO terms of biological processes targeted by DEGs included the oxidation–reduction process, the cellular nitrogen compound biosynthetic process, copper ion transmembrane transporter activity, the lipopolysaccharide biosynthetic process, mycelium development and the histidine biosynthetic process, as well as a number of up-regulated and down-regulated DEGs (Fig. 4a; Table 2). The corresponding p-values of each GO term are shown in Fig. 4b. In addition, the GO terms oxidation–reduction process and mycelium development were associated with cytochrome p450 and N-myristoyltransferase (NMT), which had been identified by inverse docking methods as potential targets.

**Table 2:** The top GO biological process terms of DEGs

GO terms	Genes_In_Term	DEGs	Up	Down	P_value
GO:0055114 (oxidation–reduction process)	777	113	60	53	2.24658E−10
GO:0044271 (cellular nitrogen compound biosynthetic process)	38	13	3	10	0.000142802
GO:0005375 (copper ion transmembrane transporter activity)	8	6	1	5	0.000153889
GO:0009103 (lipopolysaccharide biosynthetic process)	10	6	5	1	0.000705866
GO:0043581 (mycelium development)	468	60	17	43	0.000785477
GO:0000105 (histidine biosynthetic process)	8	5	0	5	0.001888677
Total	1301	198	86	112	

The KEGG pathway enrichment analysis of DEGs also provided insight into the cellular pathways associated with these DEGs. Our results indicated that there were seven pathways corresponding to the differentially expressed mRNAs that we identified (Fig. 4c, d; Table 3). Xenobiotics biodegradation and metabolism was the top pathway enriched both in number of DEGs and P values. This result suggested that this pathway may contribute to the pathogenesis of *M. oryzae*. Moreover, the PXMP4 gene was involved in both the GO term mycelium development and the xenobiotics biodegradation and metabolism pathway.

**Table 3:** The enriched KEGG pathways in the DEGs

The first level	Function	Pathway	Genes_In_Term	DEGs	Up	Down	P_value
Metabolism	Xenobiotics biodegradation and metabolism	ko00627	153	29	18	11	1.58E−06
Metabolism	Metabolism of terpenoids and polyketides	ko00903	110	18	15	3	0.001865393
Metabolism	Amino acid metabolism	ko00350	117	18	6	12	0.003563739
Cellular processes	Transport and catabolism	ko04146	108	16	14	2	0.008760919
Metabolism	Biosynthesis of other secondary metabolites	ko00960	36	8	3	5	0.015219215
Metabolism	Metabolism of cofactors and vitamins	ko00740	30	7	2	5	0.019672753
Metabolism	Amino acid metabolism	ko00330	63	10	2	8	0.042220544

### Weighted gene co-expression analysis

**Fig. 5:**
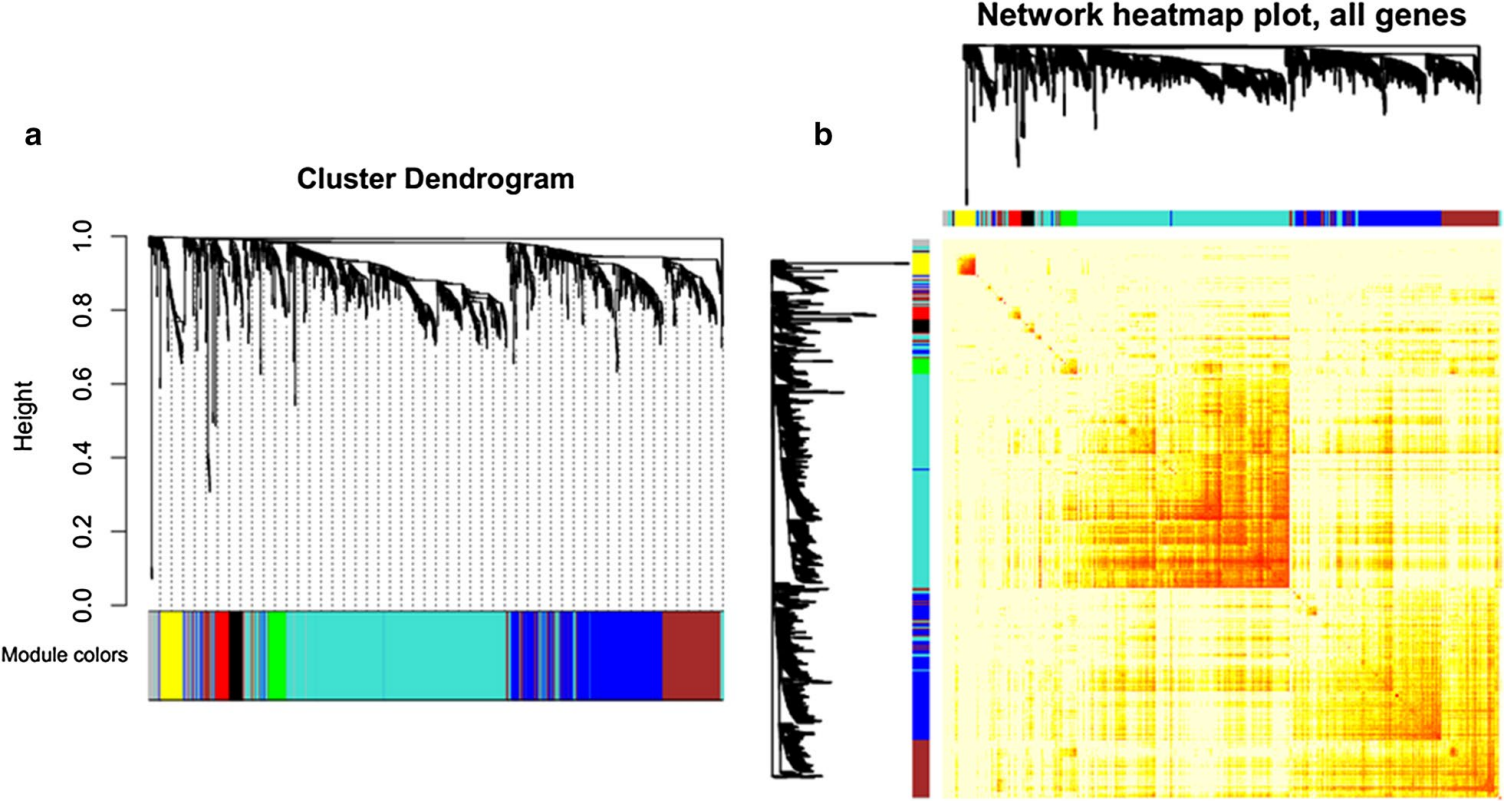
Weighted gene co-expression network analysis (WGCNA) of differentially expressed genes in *M. oryzae*. **a** The gene co-expression modules in *M. oryzae* were identified by hierarchical average linkage clustering; **b** for the dendrogram dataset, a hierarchical clustering of the topological overlap matrix was generated

To explore the regulation of M. oryzae development and metabolism and to discover hub genes involved in related biological processes, we constructed weighted gene co-expression networks based on the whole transcriptome (WGCNA method). First, seven gene co-regulation modules related with *M. oryzae* development and metabolism (correlation coefficient >0.7, P < 0.05) were extracted from the whole transcriptomic microarray results (Fig. 5a). Three modules (“brown”, “cyan” and “blue”) were subjected to further analysis.

**Fig. 6:**
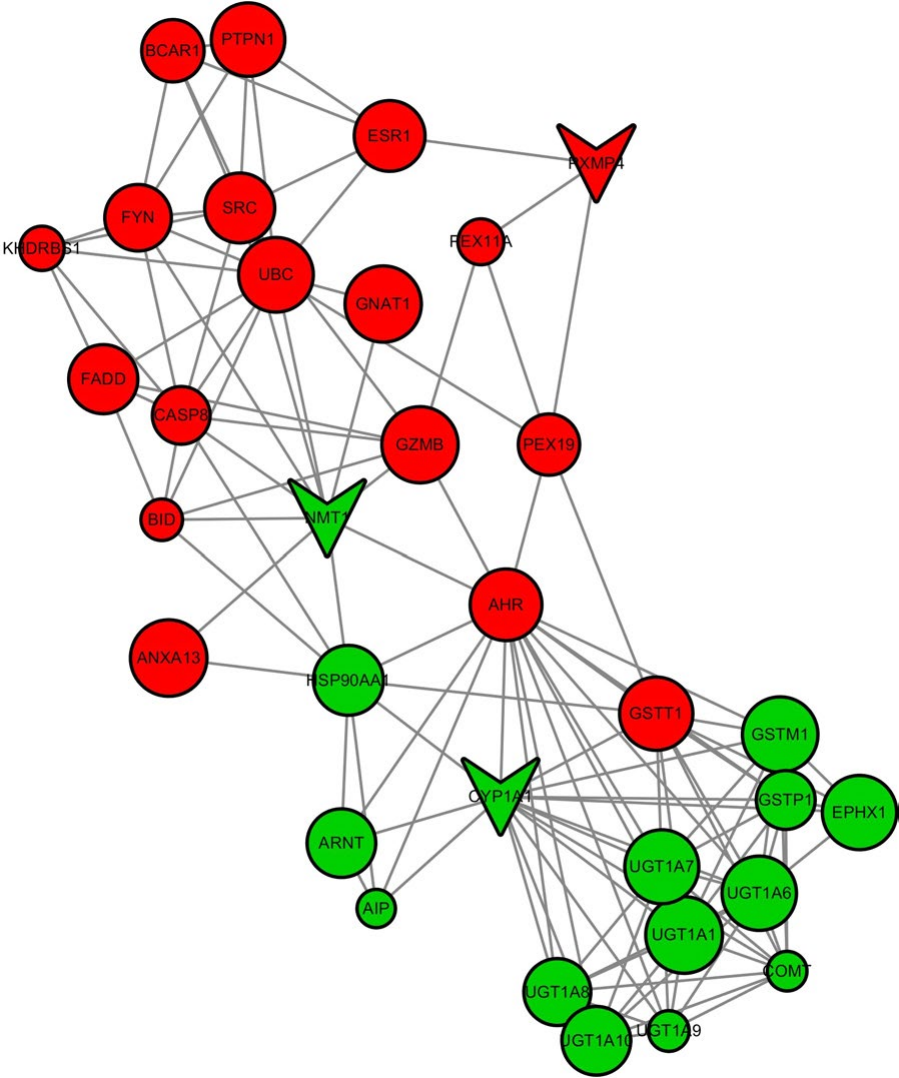
The gene co-expression networks of the three significantly DEGs. The network represents co-expression correlations between the P450, NMT, PXMP4 and their interacted DEGs. *Nodes* represent genes, *red* indicates up-regulated genes and *green* indicates down-regulated gens

We selected three genes whose differential expressions were significant in the C1 treated group to construct a gene co-expression network. These coding genes are involved in multiple biological processes, including mycelium development, oxidation and metabolism. The gene co-expression network showed that P450 was negatively correlated with PXMP4 (Fig. 6), which correlated with the oxidation–reduction process. The relationships in the modularity of the co-expression network is demonstrated via the topological overlap matrices (Fig. 5b). Interestingly, cytochrome p450, N-myristoyltransferase (NMT) and PXMP4 were involved in the hub gene modules (Fig. 6); P450, NMT and PXMP4 were differentially expressed, and the mRNA levels of β(1,3)-glucansynthase and chitin synthase were not changed and not involved in the gene co-expression network. Accordingly, we focused on P450, NMT and PXMP4 for further functional studies to elucidate its changes using experimental validation.

### qRT-PCR and Western-blot confirmation

**Fig. 7:**
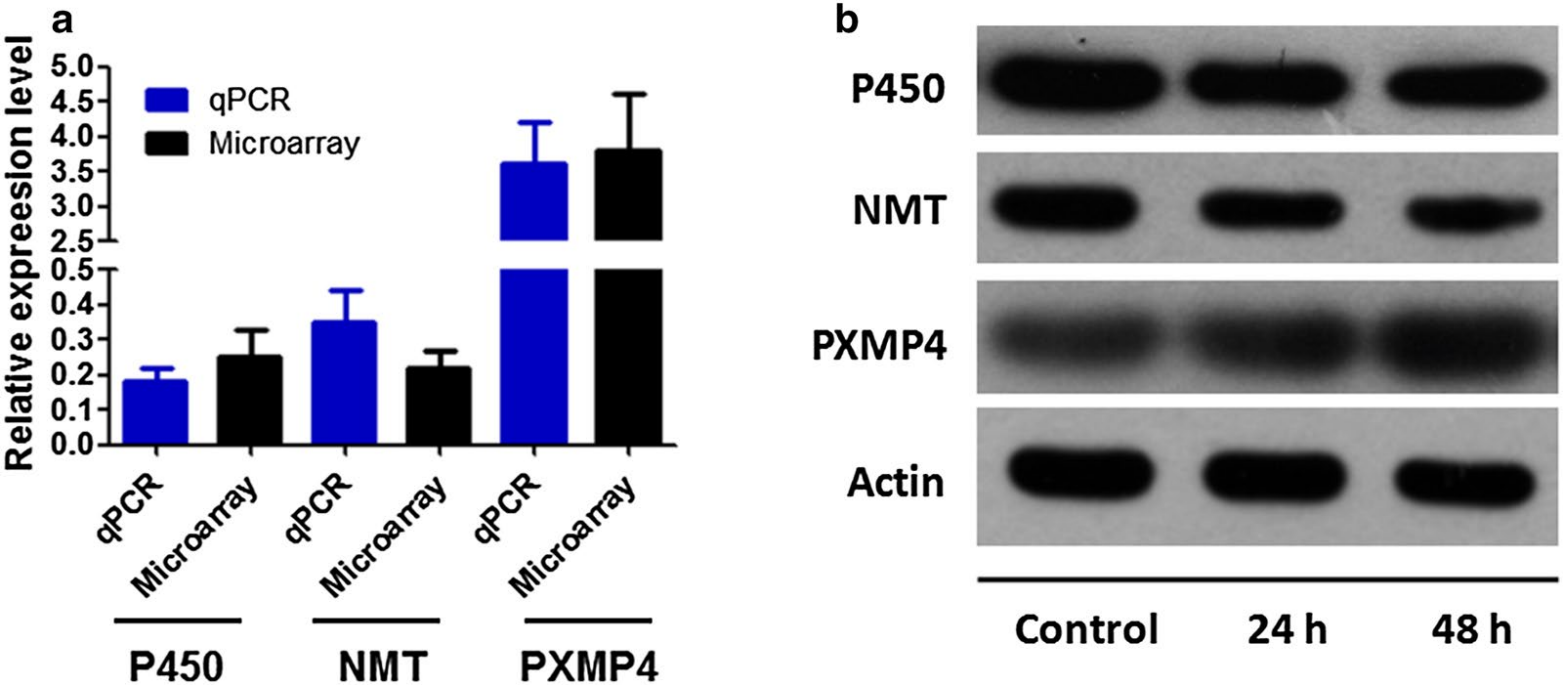
Validation of the microarray results by qRT-PCR and Western blot. **a** Values indicate the relative fold-change between the groups (drug treated vs. control group) detected by microarray (*black*) or qRT-PCR (*blue*); **b** the Western blot analysis of P450, NMT and PXMP4 protein expression levels after drug treatment

The mRNA and protein expression levels of three significantly DEGs, P450, NMT and PXMP4 were detected by qRT-PCR and Western blot analysis, respectively. Agreed with the microarray results, qRT-PCR analysis revealed that the expression of P450 and NMT was upregulated, whereas PXMP4 expression was downregulated after C1 treatment (Fig. 7a). In addition, after the compound C1 treated, we found that C1 could remarkably decrease the expression of P450 and NMT but increased PXMP4 expression with a time-dependent manner, suggesting that C1 negatively regulated xenobiotic degradation and metabolism, and potentially affected the oxidation–reduction processes of *M*. *oryzae* by regulation of peroxisome function (Fig. 7b). Therefore, the experimental validation confirmed the accuracy of the microarray results at the mRNA and protein expression levels.

## Discussion

As a novel chalcone-based phytopathogenic fungi inhibitor, C1 has good potency in protecting against the infections of various pathogens. In the current study, we found that the compound C1 could efficiently inhibit the mycelium development of *M. oryzae*, one of the most important pathogenic fungi of rice. After performing reverse-docking using three different methods, we predicted the potential target proteins of compound C1. The results indicated the possibility that cytochrome p450, N-myristoyltransferase (NMT), β(1,3)-glucansynthase and chitin synthase interact with compound C1. Furthermore, we evaluated the differential expression of mRNAs between C1-treated and control samples of *M. oryzae* using microarray analysis. Among the 13,448 embedded genes, 2008 were significantly differentially expressed. Furthermore, cytochrome p450, N-myristoyltransferase (NMT) and peroxisomal membrane protein 4 (PXMP4) were significantly differentially expressed. Collectively, these findings showed that compound C1 influenced the expression of P450, NMT and PXMP4 in *M. oryzae* and that inhibited mycelium development and exerted oxidative stress via regulation of relative downstream gene expression. In line with these results, GO analysis revealed that the DEGs were mainly enriched for GO terms associated with the response to mycelium development and oxidation–reduction processes. The KEGG pathway analysis also indicated that metabolism-associated pathways, such as xenobiotics biodegradation metabolism pathways, were the most enriched pathways. The pathway enrichment results are consistent with those of the GO term analysis, supporting the notion that mycelium development is blocked at an early stage of metabolism inhibition. Based on our data from the GO and pathway analysis, we constructed a weighted gene co-expression network to further analyze the correlations of DEGs. It has been suggested that P450, NMT and PXMP4 may be hub genes and correlate with cell death and the regulation of metabolism induced by compound C1 treatment. In general, Cytochrome P450 genes (CYPs) were key heme-proteins in primary and secondary metabolism pathways and are responsible for most oxidative/reductive reactions in the xenobiotics metabolism (Hernandez-Martinez et al. 2016; Aung et al. 2014). CYPs could detoxified and transformed various xenobiotic compounds, e.g. CYPs could converted some aromatic hydrophobic xenobiotic chemicals into non-toxic and water-soluble less metabolites via the diverse xenobiotics degradation and metabolism processes. In addition, it has been reported that *M. oryzae* and other rice blast pathogenic fungus species could tolerated high concentrations nonpolar xenobiotic chemicals. Herein, we identified that cytochrome P450 genes that might be involved in the biodegradation of xenobiotic compounds of the host plants and in sterol biosynthesis and resistance to environmental stress. It had been well known that CYPs family was one of the most abundant and diverse in *M. oryzae*. Numbers of reports had suggested that members of CYPs family to lipopolysaccharide metabolism, drug resistance, and xenobiotics metabolism by the oxidation/reduction pathways (Hernandez-Martinez et al. 2016; Huang et al. 2014; Chen et al. 2014). The CYPs family had a number of isoforms that exhibit diverse conversion capacities towards long chain alkanes, fatty acids or related molecules with different structures. In particular, it was reported that CYP proteins such as CYP1A1 could maintained signals by its trans-membrane domains and then made them localized ER proteins (Cotman et al. 2004; Szczesna-Skorupa and Kemper 2001; Szczesna-Skorupa et al. 2003). On the contrary, when such signals is lacking, proteins are transported to other regions within the cell, as was the case for the human CYP1A1. Therefore, the peroxisomal CYPs might played important roles in the metabolism of xenobiotic compounds, biosynthesis of cholesterol and hydroxylation of lipopolysaccharide, etc. In previous studies, some studies also suggested mitochondrial P450 cytochromes could be stimulated by ER P450s (Hernandez-Martinez et al. 2016; Jung and Di Giulio 2010); moreover, further studies were demanded to better understand the role of the diverse biological functions of P450s in *M. oryzae*. To confirm the above analyses, the expression of mRNAs and proteins were detected using qRT-PCR and Western blotting, respectively. These experimental results were highly consistent with the microarray and bioinformatics analyses; taken together, our findings indicate that P450 and NMT are the direct target proteins of compound C1 and that PXMP4 plays an important role in the signaling transduction networks induced by C1.

## Conclusions

In conclusion, our study suggests that the combination of transcriptomic microarray, bioinformatics analysis and weighted gene co-expression networks can be used to predict the potential targets and regulated pathways of small molecular natural product-like drugs. Moreover, we have shown that these high-throughput and computational results could be validated using various experimental methods. We have indicated an approach for profiling potential target and molecular mechanism in species with limited genomic and/or signaling pathway knowledge. We believe that this target profiling workflow can be helpful for identifying novel targets for therapeutics and for overcoming drug resistance to rare pathogens.

## Additional files

**Additional file 1.** **Table S1**, The top 100 up-regulated genes after C1 treatment; and **Table S2**, The top 100 down-regulated genes after C1 treatment.

**Additional file 2.** The probe name, expression level and annotations of transcriptome microarray results in current researches.
