# Supplementary material for: Discovery of the molecular mechanisms of the novel chalcone-based Magnaporthe oryzae inhibitor C1 using transcriptomic profiling and co-expression network analysis
Source: Springerplus. 2016 Oct 22;5(1):1851. doi: 10.1186/s40064-016-3385-9 (PMC5075332; doi:10.1186/s40064-016-3385-9)
Supplement: Supplementary file 1 — Additional file 1. Table S1, The top 100 up-regulated genes after C1 treatment; and Table S2, The top 100 down-regulated genes after C1 treatment. [file 40064_2016_3385_MOESM1_ESM.docx]

**Table S1.** The top 100 up-regulated genes after C1 treatment (p<0.05).

| ProbeName | Symbol | p-value | FC (abs) | Description |
| --- | --- | --- | --- | --- |
| A_98_P114745 | DNM | 0.000109356 | 91.42 | interferon-induced GTP-binding protein Mx2 |
| A_98_P191952 |  | 0.000411691 | 75.48 | hypothetical protein |
| A_98_P162323 |  | 0.001517579 | 60.23 | hypothetical protein |
| A_98_P127579 | adh | 0.003460584 | 48.61 | alcohol dehydrogenase |
| A_98_P197959 |  | 0.01636534 | 47.28 | hypothetical protein |
| A_98_P191984 |  | 0.007138992 | 35.16 | hypothetical protein |
| A_98_P243050 |  | 0.000291278 | 33.13 | hypothetical protein |
| A_98_P234205 | Tyr | 0.004364451 | 32.13 | tyrosinase |
| A_98_P227608 |  | 0.000300626 | 31.75 | hypothetical protein |
| A_98_P162051 |  | 4.67984E-06 | 31.00 | hypothetical protein |
| A_98_P164270 |  | 0.012810214 | 28.15 | hypothetical protein |
| A_98_P237806 |  | 0.000847876 | 26.20 | conidiation protein 6 |
| A_98_P126763 |  | 0.000455537 | 25.57 | hypothetical protein |
| A_98_P183950 |  | 0.019266827 | 24.37 | hydrophobin |
| A_98_P175563 | IL4I1 | 0.001172516 | 24.20 | amine oxidase |
| A_98_P203958 | GLT25D | 0.00142773 | 21.75 | collagen beta-1,O-galactosyltransferase |
| A_98_P143660 |  | 0.00025864 | 20.64 | cytochrome P450-like protein |
| A_98_P151905 |  | 0.001989067 | 18.31 | conidiation-specific protein 6 |
| A_98_P158476 |  | 0.011925769 | 18.28 | hypothetical protein |
| A_98_P245486 | GLP-F | 0.016336879 | 18.19 | aquaporin-9 |
| A_98_P109313 |  | 0.021936623 | 18.16 | hypothetical protein |
| A_98_P246239 |  | 0.010972224 | 17.91 | hypothetical protein |
| A_98_P109073 |  | 0.003205196 | 17.31 | phenylacetone monooxygenase |
| A_98_P101411 |  | 9.43184E-05 | 16.85 | dihydroflavonol-4-reductase |
| A_98_P105992 |  | 0.01155779 | 16.39 | hypothetical protein |
| A_98_P235061 |  | 0.008319936 | 16.03 | hypothetical protein |
| A_98_P248097 |  | 0.007038263 | 15.78 | hypothetical protein |
| A_98_P247142 |  | 0.005129988 | 15.07 | hypothetical protein |
| A_98_P155633 |  | 0.006640811 | 15.07 | hypothetical protein |
| A_98_P217109 |  | 0.022579048 | 14.95 | hypothetical protein |
| A_98_P147238 |  | 0.01989488 | 14.78 | hypothetical protein |
| A_98_P209610 |  | 0.006729439 | 14.69 | hypothetical protein |
| A_98_P154429 |  | 0.000242257 | 14.63 | hypothetical protein |
| A_98_P155109 |  | 0.005175096 | 14.55 | hypothetical protein |
| A_98_P172145 |  | 0.004065909 | 13.64 | hypothetical protein |
| A_98_P143583 |  | 0.014679601 | 13.61 | isoflavone reductase |
| A_98_P147024 |  | 0.007706828 | 13.36 | hypothetical protein |
| A_98_P135488 |  | 0.002113164 | 12.61 | hypothetical protein |
| A_98_P149514 | GLT25D | 0.001448688 | 12.60 | collagen beta-1,O-galactosyltransferase |
| A_98_P196858 |  | 0.00203665 | 12.50 | hypothetical protein |
| A_98_P138567 |  | 0.027477656 | 12.13 | hypothetical protein |
| A_98_P146985 |  | 0.00453727 | 12.12 | retinol dehydrogenase 13 |
| A_98_P159606 |  | 0.005451741 | 12.06 | hypothetical protein |
| A_98_P190596 |  | 0.002553684 | 12.05 | putative conidiation-specific protein (con-13) protein |
| A_98_P100020 |  | 0.019276906 | 12.03 | hypothetical protein |
| A_98_P237061 |  | 0.002172875 | 11.34 | polyketide synthase/peptide synthetase |
| A_98_P152549 | JMJD2, JHDM3 | 0.000258589 | 11.18 | Lysine-specific demethylase 4B |
| A_98_P238887 |  | 0.000919468 | 11.13 | NADP-dependent alcohol dehydrogenase 6 |
| A_98_P207856 |  | 0.000300426 | 11.12 | hypothetical protein |
| A_98_P117888 | glpK | 0.028121963 | 10.86 | glycerol kinase |
| A_98_P166934 |  | 0.010867739 | 10.65 | ent-kaurene oxidase |
| A_98_P171042 |  | 0.004998453 | 10.10 | hypothetical protein |
| A_98_P220993 | CDR1 | 0.00103478 | 10.07 | multidrug resistance protein |
| A_98_P174595 |  | 0.01348419 | 9.99 | hypothetical protein |
| A_98_P204070 | phoA, phoB | 0.000826596 | 9.88 | alkaline phosphatase |
| A_98_P120183 | UVI-1 | 0.005810048 | 9.84 | UVI-1 |
| A_98_P243657 |  | 0.009058556 | 9.83 | hypothetical protein |
| A_98_P127223 | SLC6A13 | 0.000241991 | 9.82 | solute carrier family 6 protein |
| A_98_P134584 |  | 0.002235405 | 9.69 | ent-kaurene oxidase, partial |
| A_98_P175385 |  | 0.00488483 | 9.65 | zinc c6 transcription factor |
| A_98_P182482 |  | 0.005192449 | 9.60 | hypothetical protein |
| A_98_P120057 |  | 0.001142222 | 9.59 | hypothetical protein |
| A_98_P124941 |  | 0.000506815 | 9.52 | cutinase |
| A_98_P220319 |  | 0.000510702 | 9.51 | hypothetical protein |
| A_98_P244212 |  | 0.029044585 | 9.30 | endoglucanase 3 |
| A_98_P115983 |  | 0.000460125 | 9.29 | hypothetical protein |
| A_98_P165249 | TPR | 0.011466083 | 9.27 | domain-containing protein |
| A_98_P112504 |  | 0.017764198 | 9.26 | alpha/beta hydrolase |
| A_98_P183673 | glpK | 0.035658803 | 9.13 | glycerol kinase |
| A_98_P202270 |  | 0.002340532 | 9.09 | hypothetical protein |
| A_98_P145326 |  | 0.008801133 | 8.88 | hypothetical protein |
| A_98_P229010 |  | 0.00999829 | 8.85 | hypothetical protein |
| A_98_P100541 |  | 0.045391124 | 8.74 | hypothetical protein |
| A_98_P148213 | BCK1 | 0.005639242 | 8.64 | serine/threonine protein kinase |
| A_98_P115397 | P450 52A5 | 0.002806234 | 8.63 | cytochrome |
| A_98_P206334 | SLD5 | 0.000561225 | 8.62 | DNA replication complex GINS protein |
| A_98_P122182 |  | 0.000216618 | 8.60 | hypothetical protein |
| A_98_P239783 |  | 0.000407368 | 8.52 | hypothetical protein |
| A_98_P231680 |  | 0.014768448 | 8.38 | hypothetical protein |
| A_98_P108090 |  | 0.00018402 | 8.34 | hypothetical protein |
| A_98_P192511 |  | 0.005488413 | 8.23 | hypothetical protein |
| A_98_P181990 |  | 0.006119798 | 8.22 | hypothetical protein |
| A_98_P117080 |  | 0.002709071 | 8.15 | 5'-nucleotidase |
| A_98_P227710 | ggt | 0.01227775 | 8.04 | putative c6 finger domain protein |
| A_98_P146162 |  | 0.001042798 | 8.04 | DNA repair protein |
| A_98_P107466 |  | 0.004106213 | 8.01 | hypothetical protein |
| A_98_P228469 |  | 0.000258499 | 7.99 | hypothetical protein |
| A_98_P119507 |  | 0.01016748 | 7.90 | hypothetical protein |
| A_98_P109500 |  | 0.002453397 | 7.90 | hypothetical protein |
| A_98_P155551 |  | 0.015089248 | 7.73 | d-lactate dehydrogenase |
| A_98_P125786 |  | 0.003551483 | 7.64 | hypothetical protein |
| A_98_P215587 |  | 0.003383999 | 7.61 | hypothetical protein |
| A_98_P194215 |  | 0.003886613 | 7.59 | covalently-linked cell wall protein |
| A_98_P152308 |  | 0.005172732 | 7.51 | hypothetical protein |
| A_98_P140054 |  | 0.004247274 | 7.40 | hypothetical protein |
| A_98_P186754 |  | 0.000306682 | 7.39 | hypothetical protein |
| A_98_P126880 |  | 0.005340555 | 7.38 | hypothetical protein |
| A_98_P104043 |  | 0.014672967 | 7.34 | hypothetical protein |
| A_98_P104537 | P450 3A24 | 0.003512968 | 7.31 | salicylate hydroxylasecytochrome |
| A_98_P159473 |  | 0.002127436 | 7.31 | hypothetical protein |

**Table S2.** The top 100 down-regulated genes after C1 treatment (p<0.05).

| ProbeName | Symbol | p | FC (abs) | Description |
| --- | --- | --- | --- | --- |
| A_98_P217919 |  | 0.034390274 | 61.96 | putative hsp40 co-chaperone protein |
| A_98_P165290 |  | 0.012123215 | 58.50 | S-adenosyl-L-methionine-dependent methyltransferase |
| A_98_P236009 |  | 0.035152066 | 46.90 | hypothetical protein |
| A_98_P145500 |  | 0.013449223 | 42.80 | hypothetical protein |
| A_98_P199490 | tdcF | 0.000143501 | 41.65 | endoribonuclease L-PSP |
| A_98_P192695 |  | 0.03382305 | 41.50 | hypothetical protein |
| A_98_P144929 |  | 0.006213017 | 39.21 | hypothetical protein |
| A_98_P222757 |  | 0.004157914 | 33.23 | ferric reductase |
| A_98_P159681 |  | 0.026934728 | 28.28 | ferric reductase |
| A_98_P179349 |  | 0.01719144 | 28.04 | hypothetical protein |
| A_98_P242794 |  | 0.024329506 | 21.54 | hypothetical protein |
| A_98_P113553 |  | 0.022285786 | 21.10 | high affinity copper transporter |
| A_98_P200975 |  | 0.000450191 | 15.27 | glycosyltransferase family 90 protein |
| A_98_P238168 | ubiG | 0.003846499 | 14.66 | methyltransferase type 11 |
| A_98_P109225 |  | 0.032843888 | 11.60 | phytanoyl-CoA dioxygenase |
| A_98_P182215 |  | 0.038828757 | 11.58 | hypothetical protein |
| A_98_P196321 | serB, PSPH | 0.000372873 | 11.56 | phosphorylcholine phosphatase |
| A_98_P103044 | cypD_E, CYP102A2_3 | 0.008936419 | 11.53 | bifunctional P-450:NADPH-P450 reductase |
| A_98_P206516 |  | 0.008582439 | 11.09 | hypothetical protein |
| A_98_P237862 |  | 0.000895656 | 10.89 | hypothetical protein |
| A_98_P124148 | CDA1 | 0.01271275 | 10.82 | chitin deacetylase 1 |
| A_98_P164650 |  | 0.039357733 | 10.41 | hypothetical protein |
| A_98_P239744 |  | 0.000511079 | 10.32 | hypothetical protein |
| A_98_P175042 |  | 0.005909681 | 10.17 | sterigmatocystin 8-O-methyltransferase |
| A_98_P126118 | TPO1 | 0.000136438 | 10.11 | cycloheximide resistance protein |
| A_98_P109335 |  | 0.003485885 | 9.74 | hypothetical protein |
| A_98_P123121 |  | 0.04742996 | 9.55 | ferric reductase like transmembrane component |
| A_98_P248130 |  | 0.00625784 | 8.99 | hypothetical protein |
| A_98_P237751 |  | 0.014788128 | 8.86 | hypothetical protein |
| A_98_P202013 |  | 0.000714282 | 8.85 | hypothetical protein |
| A_98_P239271 | mtlD | 0.000285969 | 8.81 | mannitol-1-phosphate 5-dehydrogenase |
| A_98_P236724 |  | 0.009562873 | 8.69 | hypothetical protein |
| A_98_P131887 |  | 0.005913845 | 8.65 | hypothetical protein |
| A_98_P229815 | STK16 | 0.032973956 | 8.55 | MFS transporter |
| A_98_P214919 |  | 0.00266396 | 8.49 | hypothetical protein |
| A_98_P158389 |  | 0.003069133 | 8.30 | hypothetical protein |
| A_98_P202086 | K06987 | 0.007571727 | 8.11 | carboxypeptidase 2 |
| A_98_P223312 |  | 0.003119003 | 8.04 | hypothetical protein |
| A_98_P145404 |  | 0.006805828 | 7.93 | hypothetical protein |
| A_98_P206891 | ACER, ASAH3 | 0.006668266 | 7.79 | alkaline ceramidase,neutral ceramidase |
| A_98_P235083 |  | 0.022566563 | 7.73 | hypothetical protein |
| A_98_P233765 |  | 0.007862425 | 7.70 | hypothetical protein |
| A_98_P117438 |  | 0.002239418 | 7.55 | copper transporter |
| A_98_P169780 |  | 0.022633677 | 7.47 | putative glycoside hydrolase family 79 protein |
| A_98_P251159 |  | 0.001187337 | 7.35 | hypothetical protein |
| A_98_P137032 |  | 0.000779579 | 7.32 | FAD binding domain-containing protein |
| A_98_P224221 |  | 0.008575956 | 7.30 | hypothetical protein |
| A_98_P227448 |  | 0.000382329 | 7.26 | hypothetical protein |
| A_98_P181863 |  | 0.003984693 | 7.24 | hypothetical protein |
| A_98_P205339 | dhaA | 0.0004743 | 7.12 | haloalkane dehalogenase ,twin-arginine translocation pathway signal |
| A_98_P168489 |  | 0.003739197 | 7.09 | hypothetical protein |
| A_98_P114651 |  | 0.015608936 | 6.96 | hypothetical protein |
| A_98_P175014 |  | 0.000404903 | 6.96 | hypothetical protein |
| A_98_P149480 |  | 0.02440096 | 6.70 | putative duf1445 domain protein |
| A_98_P240444 |  | 0.029660298 | 6.70 | hypothetical protein |
| A_98_P249946 |  | 0.010745111 | 6.60 | hypothetical protein |
| A_98_P138733 |  | 0.003307453 | 6.57 | hypothetical protein |
| A_98_P140745 |  | 0.010612227 | 6.50 | hypothetical protein |
| A_98_P175208 |  | 0.027308032 | 6.37 | hypothetical protein |
| A_98_P192164 |  | 0.00804658 | 6.27 | hypothetical protein |
| A_98_P101096 |  | 0.003725443 | 6.22 | hypothetical protein |
| A_98_P164923 |  | 0.002573615 | 6.12 | hypothetical protein |
| A_98_P230917 |  | 0.001104155 | 6.09 | hypothetical protein |
| A_98_P132691 |  | 0.03214358 | 6.09 | hypothetical protein |
| A_98_P177212 |  | 0.002330788 | 5.97 | diaminopropionate ammonia-lyase |
| A_98_P249165 |  | 0.001687006 | 5.94 | hypothetical protein |
| A_98_P129128 | MAS3 | 0.000443352 | 5.79 | MAS3 protein |
| A_98_P209519 |  | 0.006427324 | 5.77 | hypothetical protein |
| A_98_P234952 |  | 0.001567419 | 5.76 | hypothetical protein |
| A_98_P244813 |  | 0.034499764 | 5.76 | hypothetical protein |
| A_98_P122344 |  | 0.003307388 | 5.76 | hypothetical protein |
| A_98_P108115 |  | 0.017197715 | 5.71 | hypothetical protein |
| A_98_P225370 |  | 0.014013547 | 5.70 | hypothetical protein |
| A_98_P142233 |  | 0.003807995 | 5.67 | hypothetical protein |
| A_98_P205439 |  | 0.016387839 | 5.67 | hypothetical protein |
| A_98_P159827 | cfa | 0.007527839 | 5.56 | putative fatty acid methyltransferase |
| A_98_P194819 | K01436 | 0.005639636 | 5.53 | N-acyl-L-amino acid amidohydrolase |
| A_98_P129400 |  | 0.035736065 | 5.53 | hypothetical protein |
| A_98_P110155 |  | 0.006515341 | 5.49 | hypothetical protein |
| A_98_P209658 | ureG | 0.002516371 | 5.46 | urease accessory protein-like protein |
| A_98_P134914 |  | 0.039490215 | 5.43 | hypothetical protein |
| A_98_P223090 |  | 0.004904793 | 5.34 | hypothetical protein |
| A_98_P127824 |  | 0.002218039 | 5.29 | F-box domain-containing protein |
| A_98_P130587 |  | 0.000178555 | 5.24 | hypothetical protein |
| A_98_P241238 |  | 0.001387086 | 5.23 | hypothetical protein |
| A_98_P147921 |  | 0.000988232 | 5.21 | calcium-transporting ATPase 1 |
| A_98_P224176 |  | 0.003160631 | 5.20 | variant |
| A_98_P194414 | RAD50 | 5.77587E-05 | 5.20 | putative mfs multidrug transporter protein |
| A_98_P206107 | SLC2A13, ITR | 0.008408918 | 5.05 | lactose permease |
| A_98_P197625 | RP-L36 | 0.015927063 | 4.99 | CFEM domain-containing protein |
| A_98_P212929 |  | 0.011308159 | 4.99 | hypothetical protein |
| A_98_P141329 |  | 0.000807685 | 4.99 | hypothetical protein |
| A_98_P224482 | GLT25D | 0.002718069 | 4.95 | collagen beta-1,O-galactosyltransferase |
| A_98_P197616 | SOU1 | 0.007813046 | 4.92 | sorbose reductase |
| A_98_P184361 |  | 0.004879726 | 4.90 | Myb-like transcription regulator |
| A_98_P129391 |  | 0.000813891 | 4.89 | hypothetical protein |
| A_98_P136520 |  | 0.011188434 | 4.87 | putative glyoxylate reductase protein |
| A_98_P176731 | DAL | 0.014131423 | 4.84 | MFS transporter, ACS family, allantoate permease |
| A_98_P110680 |  | 0.009419203 | 4.84 | hypothetical protein |
| A_98_P148000 |  | 0.031780105 | 4.79 | hypothetical protein |
